# The Correlation between Apparent Diffusion Coefficient and Tumor Cellularity in Patients: A Meta-Analysis

**DOI:** 10.1371/journal.pone.0079008

**Published:** 2013-11-11

**Authors:** Lihua Chen, Min Liu, Jing Bao, Yunbao Xia, Jiuquan Zhang, Lin Zhang, Xuequan Huang, Jian Wang

**Affiliations:** 1 Department of Radiology, Southwest Hospital, Third Military Medical University, Chongqing, China; 2 Department of Radiology, Taihu Hospital, Wuxi, China; 3 Department of Administration Office, Yangpu District Center for Disease Control and Prevention, Shanghai, China; 4 Wuxi center for disease control and prevention, Wuxi, Jiangsu, China; UCSF, United States of America

## Abstract

**Objective:**

To perform a meta-analysis exploring the correlation between the apparent diffusion coefficient (ADC) and tumor cellularity in patients.

**Materials and Methods:**

We searched medical and scientific literature databases for studies discussing the correlation between the ADC and tumor cellularity in patients. Only studies that were published in English or Chinese prior to November 2012 were considered for inclusion. Summary correlation coefficient (r) values were extracted from each study, and 95% confidence intervals (CIs) were calculated. Sensitivity and subgroup analyses were performed to investigate potential heterogeneity.

**Results:**

Of 189 studies, 28 were included in the meta-analysis, comprising 729 patients. The pooled r for all studies was −0.57 (95% CI: −0.62, −0.52), indicating notable heterogeneity (*P*<0.001). After the sensitivity analysis, two studies were excluded, and the pooled r was −0.61 (95% CI: −0.66, −0.56) and was not significantly heterogeneous (*P* = 0.127). Regarding tumor type subgroup analysis, there were sufficient data to support a strong negative correlation between the ADC and cellularity for brain tumors. There was no notable evidence of publication bias.

**Conclusions:**

There is a strong negative correlation between the ADC and tumor cellularity in patients, particularly in the brain. However, larger, prospective studies are warranted to validate these findings in other cancer types.

## Introduction

Diffusion-weighted imaging (DWI), which tracks the microscopic rate of water diffusion within tissues, is a magnetic resonance imaging (MRI)-based technique that has provided a new means of tracking tumor progression and response to treatment. The apparent diffusion coefficient (ADC) typically replaces the diffusion coefficient as a diffusion index in biological systems because the latter depends on factors beyond Brownian motion, such as microcirculation. Because it provides information about tissue cellularity and the integrity of cell membranes [1], DWI has benefits over traditional anatomical MRI techniques.

Generally, tumor cell proliferation increases tumor cellularity, whereas tumor cell apoptosis reduces tumor cellularity. Tumor cellularity and the shape of the extracellular space affect diffusion. The diffusivity of water molecules is restricted in environments of high cellularity because this cellularity reduces the ratio of extracellular to intracellular space in a given area of tissue [2], [3]. Studies conducted *in vitro* [4], [5] and in animal models [6], [7] show that the ADC is inversely correlated with tumor cellularity. The hypothesis that the ADC is also inversely correlated with tumor cellularity in patients makes DWI a widely applicable method for differentiating benign from malignant lesions, monitoring the treatment response after chemotherapy or radiation, and detecting recurrent cancer [8]. However, the results of studies attempting to verify this hypothesis are controversial; certain studies confirmed a notable negative correlation between the ADC and tumor cellularity [9]–[32], whereas other studies presented negative [33]–[37] or even inverse results [35], [36]. In addition, the sample sizes of these studies were small.

Therefore, we performed the present meta-analysis to explore the correlation between the ADC and tumor cellularity in patients and to investigate variations in the methods used in previous studies.

## Materials and Methods

### Literature Search

Two independent observers searched the following databases in September 2012: PubMed, Embase, the Cochrane Library, and the China National Knowledge Infrastructure (CNKI). The databases were searched using the terms “diffusion-weighted imaging OR DWI,” AND “cell density OR cellularity OR cell count OR cell number,” AND “apparent diffusion coefficient OR ADC.” The search was limited to publications written in English or Chinese to match our translation capacity. We searched publications published prior to and including November 2012. The reference lists of all retrieved articles were manually cross-checked.

### Selection of Articles

Articles were selected for inclusion if they met the following criteria: (a) investigation of the relationship between the ADC and tumor cellularity; (b) inclusion of patients with tumors, which could include patients with benign conditions as long as most patients in the sample had cancer; (c) identification and characterization of tumors, both benign and malignant by histopathologic analysis; and (d) publication as a full paper in a peer-reviewed scientific journal.

The following studies were excluded: (a) multiple reports published on the same study population (in this case, the publication that included the most details and/or that was most recently published was chosen); (b) studies *in vitro* or in animal models; (c) studies analyzing the relationship between the ADC and tumor cellularity with treatment; and (d) review articles, letters, comments, case reports, and unpublished articles (abstracts only).

### Quality Assessment and Data Extraction

The methodological quality of the included studies was independently assessed by two observers using the Quality Assessment of Diagnostic Studies (QUADAS) instrument, a quality assessment tool specifically developed for systematic reviews of diagnostic accuracy studies [38], [39]. The information extracted from each publication, in the form of a table, included the following: authors, the nation of origin, the year of publication, the number and ages of the patients, b values, techniques, MRI field strength, vendors, Pearson or Spearman correlation coefficient (r), and the index used to characterize the ADC (average or minimum expression). Disagreements between the two reviewers were resolved by a majority opinion after a third reviewer assessed all involved items.

The correlation coefficients were calculated from a scatter plot of the ADC and tumor cellularity for cases in which the correlation coefficients were not reported. First, Engauge Digitizer software (free software downloaded from http://sourceforge.net) was used to convert the scatter plots into coordinates. In this way, we obtained the ADC values and tumor cellularity indirectly. Second, SPSS software was used to calculate the correlation coefficients. Because certain variables in the original studies were log-transformed before analysis, Spearman correlation coefficients were used for the meta-analyses. Spearman correlation coefficients are unaffected by monotonic transformations, such as a logarithmic transformation. The published Pearson correlation coefficients were converted into Spearman correlation coefficients [40], [41]. The sampling distribution of Spearman correlation coefficients is problematic because the standard error (SE) depends on the value of the correlation coefficient. Thus, a Fisher transformation was used to convert each correlation coefficient into an approximately normal distribution.

### Meta-Analysis

After appropriate conversion, data from the various studies were combined using random effects meta-analyses [42]. The heterogeneity of the r values between studies was determined by calculating the Q statistic, derived from the chi-square test, and the inconsistency index (I^2^) [43], [44]. A *P*-value <0.05 or an I^2^ value >50% suggested heterogeneity [45]. If notable heterogeneity was detected, a sensitivity analysis was performed for all studies to further investigate the study heterogeneity.

In a subgroup analysis, studies were stratified by the following: (a) tumor type, (b) the index of the average ADC (meanADC) or minimum ADC (minADC), (c) magnetic field strength (1.5 or 3.0 T), (d) a b value ≥1000 s/m^2^ or <1000 s/m^2^, (e) design (prospective or retrospective), (f) patient age (adult or child), (g) vendors, and (h) the definition of tumor cellularity (cell count, cell density, or nuclear-to-cytoplasmic ratio).

The results of Begg’s funnel plot (*P* = 0.103) showed no evidence of notable publication bias (Fig. 6).

The presence of publication bias was visually assessed using a funnel plot. Statistical manipulation was performed with the software STATA version 11 (Stata Corporation, College Station, TX, USA).

**Figure 1:**
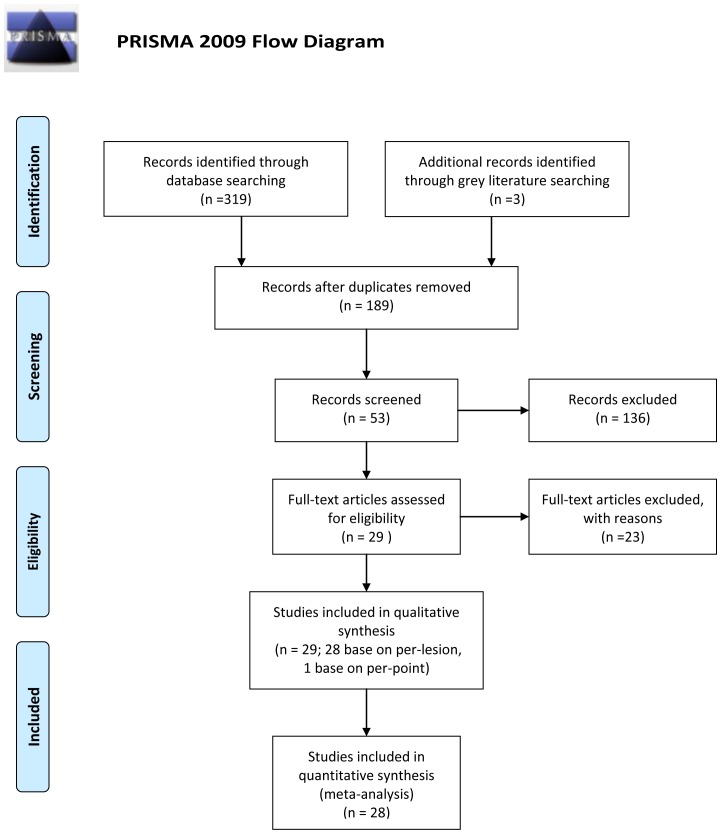
Flowchart of the study selection process.

The Preferred Reporting Items for Systematic Reviews and Meta-Analyses statement (PRISMA) [46] was used to improve the reporting of our research (Fig. 1 and Checklist S1).

## Results

**Figure 2:**
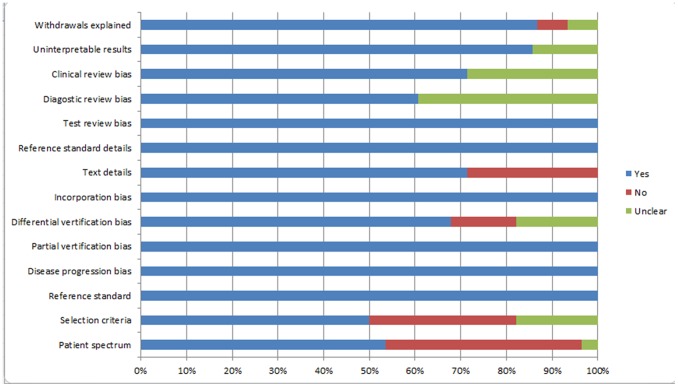
Methodological quality of the 28 studies.

The search initially yielded 189 potential literature citations (Fig. 1). In total, 136 of these studies were immediately excluded after reviewing the abstracts due to non-relevance (n = 104), tumor treatment (n = 17), *in vitro* experiments or animal model use (n = 12), or publication in languages other than English or Chinese (n = 3). After reading the full texts of the remaining 53 articles, 24 were excluded due to either a lack of sufficient information to calculate the correlation coefficients or the use of *in vitro* or animal model-based experiments. In the extracted 29 studies, one study [37] was performed based on a per-point analysis of biopsies, whereas the other included studies were all based on per-lesion analyses. As the sample sizes for the data reported on a per-point basis were too small, the data analysis in this study was performed only on a per-lesion basis. Finally, 28 published studies (English language, n = 27; Chinese language, n = 1) fulfilled our inclusion and exclusion criteria, and a total of 30 experiments were analyzed because two studies [24], [25] included two experiments. The median number of patients per study was 25.7 (range 7–124), with a total of 729 patients. The most studied tumor location was the brain, for which there were 13 studies. The extracted data from these individual studies are summarized in Table 1. The quality assessment was moderate in the 28 studies according to the QUADAS items, and the distribution of the study design is shown in Fig. 2.

**Table 1:** Characteristics of the included studies.

Study	Year	Nation	N	Tumor	Age	Design	Field	Index	b value^a^	r^b^
Sugahara [26]	1999	Japan	20	brain	Adult	prospective	1.5 T	minADC	1200	−0.75
Gupta [20]	2000	USA	18	brain	Adult	prospective	1.5 T	meanADC	940	−0.65^c^
Gauvain [19]	2001	USA	12	brain	Children	retrospective	1.5 T	meanADC	1012	−0.67
Kono [25]	2001	Japan	17	brain	Adult	retrospective	1.5 T	meanADC	1000	−0.75
	2001	Japan	18	brain	Adult	retrospective	1.5 T	meanADC	1000	−0.65
Guo A [32]	2002	USA	28	brain	Adult	retrospective	1.5 T	meanADC	1000	−0.46
Guo Y [14]	2002	China	47	breast	Adult	retrospective	1.5 T	meanADC	1000	−0.51
Chen [10]	2005	China	34	brain	Adult	retrospective	1.5 T	meanADC	1000	−0.52
Hayashida [22]	2006	Japan	13	brain	Adult	retrospective	1.5 T	meanADC	1000	−0.68
Plank [30]	2007	Austria	8	spinal	Adult	retrospective	1.5 T	meanADC	700	−0.64^d^
Matoba [27]	2007	Japan	9	lung	Adult	prospective	1.5 T	meanADC	577	−0.75
Humphries [28]	2007	USA	19	various	Children	prospective	1.5 T	meanADC	1000	−0.72^c^
Zelhof [15]	2008	UK	38	prostate	Adult	prospective	3.0 T	meanADC	500	−0.48
Hatakenaka [30]	2008	Japan	124	breast	Adult	prospective	1.5 T	meanADC	1000	−0.65^c^
Manenti [21]	2008	Italy	27	renal	Adult	retrospective	3.0 T	meanADC	500	−0.71
Yoshikawa [33]	2008	Japan	27	breast	Adult	retrospective	1.5 T	meanADC	800	0.05
Woodhams [13]	2009	Japan	15	breast	Adult	retrospective	1.5 T	meanADC	1500	−0.74
Wang [16]	2009	China	38	prostate	Adult	retrospective	1.5 T	meanADC	500	−0.63
Yamashita [31]	2009	Japan	26	brain	Adult	retrospective	1.5 T	minADC	1000	−0.69
Gibbs [9]	2009	UK	20	prostate	Adult	prospective	3.0 T	meanADC	500	−0.68
Kikuchi [11]	2009	Japan	10	brain	Adult	retrospective	1.5 T	minADC	1000	−0.66
Jenkinson [32]	2010	UK	17	brain	Adult	retrospective	1.5 T	both	1000	0.04
Ellingson [18]	2010	USA	17	brain	Adult	retrospective	1.5 T	meanADC	1000	−0.88^c^
Barajas [23]	2010	USA	18	brain	Adult	retrospective	1.5 T	meanADC	1000	−0.52
Kyriazi [24]	2010	UK	8	ovarian	Adult	retrospective	1.5 T	meanADC	1050	−0.77
	2010	UK	7	omental	Adult	retrospective	1.5 T	meanADC	1050	−0.72
Wang [31]	2011	USA	18	pancreas	Adult	retrospective	1.5 T	meanADC	600	−0.35
Goyal [12]	2012	India	36	renal	Adult	retrospective	1.5 T	meanADC	500	−0.31
Doskaliyev [17]	2012	Japan	24	brain	Adult	retrospective	3.0 T	meanADC	1000	−0.58
Ginat [29]	2012	USA	18	skull	Adult	retrospective	1.5 T	meanADC	1000	−0.58

minADC = measurement of minimum ADC value, meanADC = measurement of average ADC value.

a The unit of the b value is s/m^2^.

b r = Spearman correlation coefficient.

c r values were calculated based on r^2^ values.

d The r value was calculated indirectly from the scatter diagram.

All studies provided data suitable for a meta-analysis. For four studies [18], [20], [28], [30], the r values were calculated based on the r^2^ values provided in the papers, and the graphical representations were examined to determine the sign. For one study [33], the r value was calculated indirectly from the scatter diagram provided in the paper.

**Figure 3:**
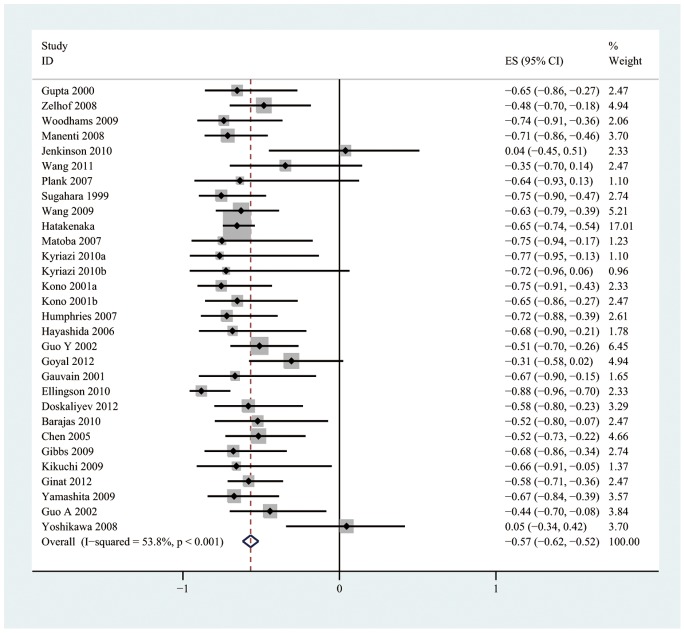
Forest plots of the summary correlation coefficient (r) with corresponding 95% CIs for the correlation between the ADC value and tumor cellularity in patients from all eligible studies.

**Figure 4:**
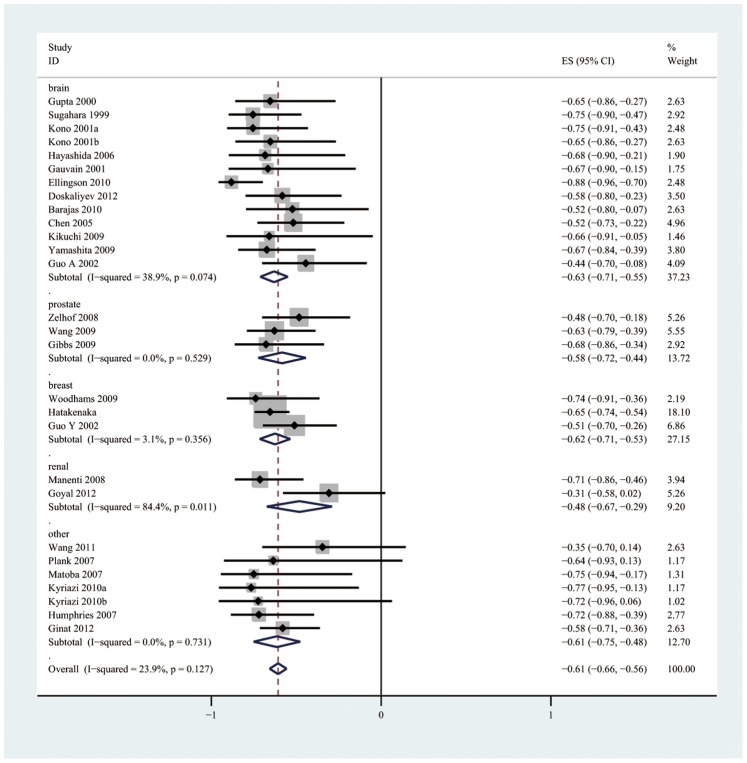
Forest plots of the pooled r with corresponding 95% CIs after two studies were excluded following a sensitivity analysis and forest plots of the subgroup analysis based on tumor type.

The pooled r for all studies (Fig. 3) was −0.57 (95% CI: −0.62, −0.52) and exhibited notable heterogeneity (I^2^ = 53.8%, *P*<0.001). After a sensitivity analysis of the overall group of studies, two studies [35], [36] that were considered to be homogeneous were excluded. The pooled r after the two homogeneous studies were excluded (Fig. 4) was −0.61 (95% CI: −0.66, −0.56) and was not notably heterogeneous (I^2^ = 23.9%, *P = *0.127).

**Figure 5:**
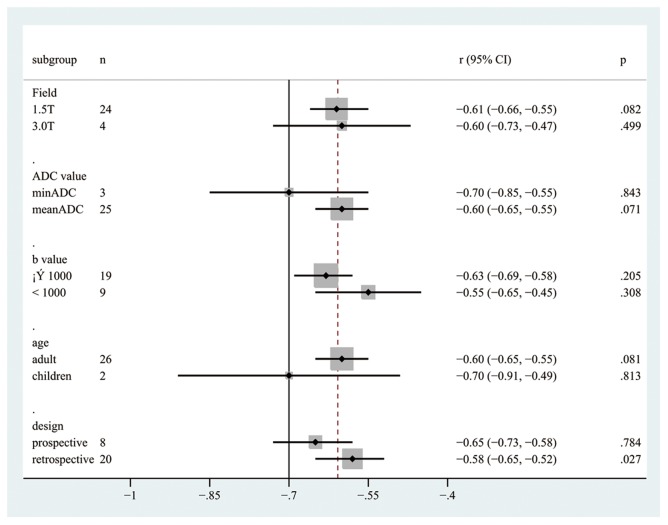
The pooled r with corresponding 95% CIs for the subgroup analysis based on magnetic field strength, the index of the ADC value, the b value, age, and design.

There were no significant differences between all subgroups. The pooled r values estimated for the different subgroups are presented in Table 2 and Fig. 5.

**Figure 6:**
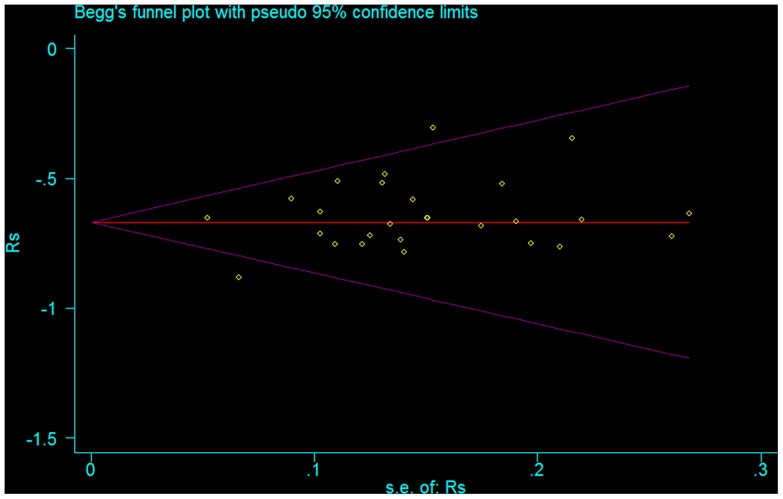
The funnel plot of the publication bias. The result is suggestive of an indistinctive small study bias (*P* = 0.103).

**Table 2:** Sensitivity estimates for each subgroup.

Subgroup	No. ofexperiments	r (95% CI)	I^2^	P value
Definition^a^				
Cell count	3	−0.61(−0.78, −0.45)	38.5%	0.197
Cell density	13	−0.62(−0.70, −0.54)	40.4%	0.064
N/C ratio	12	−0.60(−0.67, −0.53)	0.0%	0.450
Vendor^a^				
GE	13	−0.55(−0.63, −0.47)	63.5%	0.001
Philips	2	−0.70(−0.89, −0.50)	0.0%	0.776
Siemens	9	−0.65(−0.73, −0.58)	0.0%	0.865
No mention	4	−0.66(−0.79, −0.58)	0.0%	0.702
Tumor type^a^				
Brain	13	−0.62(−0.71, −0.54)	41.7%	0.057
Prostate	3	−0.58(−0.72, −0.44)	0.0%	0.529
Breast	3	−0.62(−0.71, −0.53)	3.1%	0.356
Renal	2	−0.48(−0.67, −0.29)	84.4%	0.011
Other^b^	7	−0.61(−0.75, −0.48)	0.0%	0.731

N/C ratio = nuclear-to-cytoplasmic ratio.

a There are no significant differences between the subgroups of tumors.

b Includes tumors of the lung, ovaries, omentum, skull, pancreas, spine, and various other locations.

## Discussion

The aims of our meta-analysis were to explore the correlation between the ADC and tumor cellularity and to investigate variations in the methods of clinical application. We excluded studies performed *in vitro* and in animal models because many factors that affect diffusion must be controlled in both. We also excluded therapeutic studies to simplify our analysis and to increase its accuracy. Additionally, several effective tumor treatments have been shown to increase the ADC [47], whereas others result in a reduction [47]. The tumor ADC has also been shown to change nonlinearly over the course of treatment [28], [48].

Our meta-analysis of published studies showed that there was a significant negative correlation between the ADC and tumor cellularity in patients. Our findings provide evidence that DWI can be used as a biomarker for tumor cellularity. Compared with benign lesions, malignant tumors have larger nuclei, richer stroma, and higher cell counts, which lead to greater cellularity. By measuring the ADC, DWI can be used to distinguish benign from malignant tumors. In general, any effective pharmacologic or radioactive treatment that causes necrosis or cellular lysis will lead to less cellularity. A decrease in the number of tumor cells in response to treatment obviously precedes size change; therefore, DWI may be an early biomarker for predicting treatment outcomes, monitoring the early treatment response, and detecting recurrent cancer.

There was noticeable heterogeneity in all of the included studies, so we investigated the sources of this heterogeneity. A sensitivity analysis identified two studies that caused heterogeneity, which were excluded. One of the two studies [35] focused on oligodendroglial tumors because oligodendroglial tumors with 1p/19q loss are more likely to have a low ADC than tumors with intact 1p/19q. In the other study [36], both invasive ductal carcinoma and noninvasive ductal carcinoma were analyzed together, and the authors speculated that the ADC may be affected not only by cancer cellularity but also by histological type. Generally, the values for diffusion found in most tumors have been attributed to the tumors’ cellular density; however, this concept remains controversial because diffusivity is influenced by other histological characteristics, such as fibrosis, the shape and size of the intercellular spaces, and glandular structure (as in well-differentiated adenocarcinomas). We also performed a subgroup analysis based on the histological type. The result showed no notable variation between the subgroups based on tumor type. However, we observed that the correlation between the ADC and tumor cellularity differed between histological types, with correlation coefficients ranging from −0.79 (liver tumor, n = 1) to −0.35 (pancreatic endocrine tumor, n = 1). We believe that sample sizes large enough for comparison could be a source of heterogeneity.

Other sources of heterogeneity may be present, including the technical characteristics of the DWI scanning and measurements that were compared between the reviewed studies. Indeed, the implementation of scanning protocols and measurement by different companies varies significantly. Moreover, there is divergent nomenclature among the vendors for the implementation of DWI [8]. It is also clear that variations in the b value exist and that there is no consensus on the measurement index of the ADC or the magnetic field strengths. Our subgroup analysis indicated that none of these factors contributed to the observed heterogeneity. The validations among vendors and the magnetic field strengths (1.5 and 3.0 T) were nearly identical. However, the application of the index minADC and a high b value (b value ≥1000 s/m^2^) may be more related to tumor cellularity. We recommend specific experiments to further investigate variations in these methods. If confirmed, our finding would provide evidence for establishing clinical DWI acquisition and analysis guidelines.

Certain inherent limitations existed in our study design and should be considered when interpreting our results. First, the number of patients in several of the included studies was relatively small, and the number of patients included for each organ was relatively small, which may reduce the strength of the conclusions in this paper. Second, our meta-analysis was based only on published studies, which tend to report positive or significant results; studies with insignificant or negative results are often rejected or are not submitted. This feature may have led to a publication bias, which tends to overestimate results. However, it is likely that the quality of the data reported in articles accepted for publication in peer-reviewed journals is superior to the quality of unpublished data [49]. In addition, this review was restricted to articles published in English or Chinese because other languages, such as Cabada [50], could not be translated by the study authors, which may have introduced bias.

In conclusion, despite the limitations of our meta-analysis, all currently available evidence supports a strong negative correlation between the ADC and tumor cellularity in patients, particularly in brain, prostate, breast, and renal tumors. However, larger, prospective studies are warranted to validate these findings in other cancer types. Future validation studies of DWI will likely benefit from the following: (a) the application of the index to both the minADC and the meanADC, (b) the inclusion of high and low b values, and (c) the establishment of specific guidelines for performing and analyzing standard clinical DWI scans.

## Supporting Information

Checklist S1 PRISMA 2009 checklist.
(DOC)
